# Creation of Sub-diffraction Longitudinally Polarized Spot by Focusing Radially Polarized Light with Binary Phase Lens

**DOI:** 10.1038/srep38859

**Published:** 2016-12-12

**Authors:** An-ping Yu, Gang Chen, Zhi-hai Zhang, Zhong-quan Wen, Lu-ru Dai, Kun Zhang, Sen-lin Jiang, Zhi-xiang Wu, Yu-yan Li, Chang-tao Wang, Xian-gang Luo

**Affiliations:** 1 Key Laboratory of Optoelectronic Technology and Systems (Chongqing University), Ministry of Education, and Key Disciplines Lab of Novel Micro-nano Devices and System Technology, Chongqing University, 173 Shazheng Street, Shapingba, Chongqing 400044, China; 2 National Center for Nanoscience and Technology, No. 11 Zhong Guan CunBei Yi Tiao, Beijing 100190, China; 3 State Key Laboratory of Optical Technologies on Nano-Fabrication and Micro-Engineering, Institute of Optics and Electronics, Chinese Academy of Science, P.R. Box 350, Chengdu 610209, China

## Abstract

The generation of a sub-diffraction longitudinally polarized spot is of great interest in various applications, such as optical tweezers, super-resolution microscopy, high-resolution Raman spectroscopy, and high-density optical data storage. Many theoretical investigations have been conducted into the tight focusing of a longitudinally polarized spot with high-numerical-aperture aplanatic lenses in combination with optical filters. Optical super-oscillation provides a new approach to focusing light beyond the diffraction limit. Here, we propose a planar binary phase lens and experimentally demonstrate the generation of a longitudinally polarized sub-diffraction focal spot by focusing radially polarized light. The lens has a numerical aperture of 0.93 and a long focal length of 200λ for wavelength λ = 632.8 nm, and the generated focal spot has a full-width-at-half-maximum of about 0.456λ, which is smaller than the diffraction limit, 0.54λ. A 5λ-long longitudinally polarized optical needle with sub-diffraction size is also observed near the designed focal point.

There is a growing interest in tight focusing of radially polarized light, due to its unique property of a strong longitudinal and nonpropagating electric field in the focal region. In the focal spot, compared with the radial polarization component, the longitudinal electrical field is dominant and makes no contribution to the energy flow along the optical axis, which is important in metallic particle trapping^1^, particle acceleration^2^, and tip-enhanced Raman spectroscopy^3^. Furthermore, theoretical works have predicted that focusing radially polarized light can create a longitudinal electric field component with a smaller focal spot size than linearly polarized and circularly polarized beams. This property is of great importance in applications such as microscopes^4^,^5^ and optical data storage^6^. Generation of a sub-wavelength focal spot with a strong longitudinal electric field has been theoretically proposed using various methods, such as use of a parabolic mirror^7^, the combination of a binary optical phase filter and a conventional high-numerical-aperture optical lens^8^,^9^,^10^,^11^,^12^, a negative-index grating lens^13^, a 4π high-numerical-aperture focusing system^14^, and absorbance modulation^15^. Compared with conventional optical lenses, planar lenses are attractive because they are small, lightweight, and easily integrated. Theoretical investigations have been conducted into the tight focusing of radially polarized light using a planar plasmonic metalens based on the parabolic phase profile^16^, and the resulting focal spot size was still diffraction-limited. Although super-resolution can be achieved by near-field optics^17^,^18^, super-oscillation provides a promising approach to realize far-field sub-diffraction focusing and imaging. Many planar diffractive lenses have been proposed for focusing and imaging beyond the diffraction limit^19^,^20^,^21^,^22^,^23^,^24^,^25^,^26^,^27^. Although metasurfaces^26^,^28^ have been reported for focusing purposes, their amplitude transmission rate is very low and they are difficult to be fabricated in visible wavelength range. Recently, super-oscillation planar lenses based on quasi-continuous amplitude modulation^29^,^30^ and a binary amplitude-phase mask^31^ have been demonstrated for sub-diffraction focusing of linearly polarized light. A binary amplitude-phase-mask-based super-oscillation lens was reported for the super-oscillatory focusing of circularly polarized waves with an ultra-long focal length and small numerical aperture (NA)^32^. For this paper, we experimentally demonstrated the generation of a sub-diffraction longitudinally polarized spot by focusing radially polarized light with a planar binary phase lens.

## Materials and Methods

### Binary phase lens design

The radially polarized wave can be generated by an s-wave plate, which converts a linearly polarized Gaussian beam into a radially polarized beam in the Laguerre-Gaussian profile. The resulting electrical field can be described by equation 1, where E_0_ is the incident electrical field amplitude, w_0_ is the beam waist size, z_0_ = πw_0_^2^/λ is the Rayleigh range, R(z) = z[1 + (z_0_/z)^2^] is the radius of curvature, w(z) = w_0_[1 + (z/z_0_)^2^]^1/2^ is the beam width at z, and k = 2*π/*λ is the wavenumber.


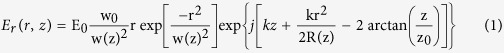


According to the vectorial angular spectrum theory^23^, for a radially polarized wave impinging on a circularly symmetrical planar lens, its diffracted electrical field on the focal plane at z = z_*f*_ can be expressed by equations (2) in cylindrical coordinate, where E_r_, E_φ_, and E_z_ are the radial, azimuthal, and longitudinal polarization components of the diffracted electrical field, respectively; *r* and ρ are radial coordinates in the spatial and frequency domains, respectively; g(*r*) = E_r_(*r*, z_*l*_) is the complex amplitude distribution of the radially polarized electrical field on the lens input surface, z_*l*_ being the position of the lens input surface; t(*r*) is the complex amplitude transmittance function of the lens; J_0_ and J_1_ are the zero- and first-order Bessel functions, respectively; and q(ρ) = (1/λ^2^−ρ^2^)^1/2^ is the frequency component in the propagation direction. According to the equations, it is clear that, besides the radial polarization electrical component, the diffracted electrical field also includes a longitudinal polarization, which plays a key role in the focusing, as shown later. Notice also that the azimuthal polarization is zero.


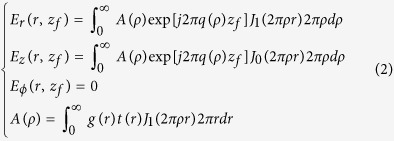


**Figure 1:**
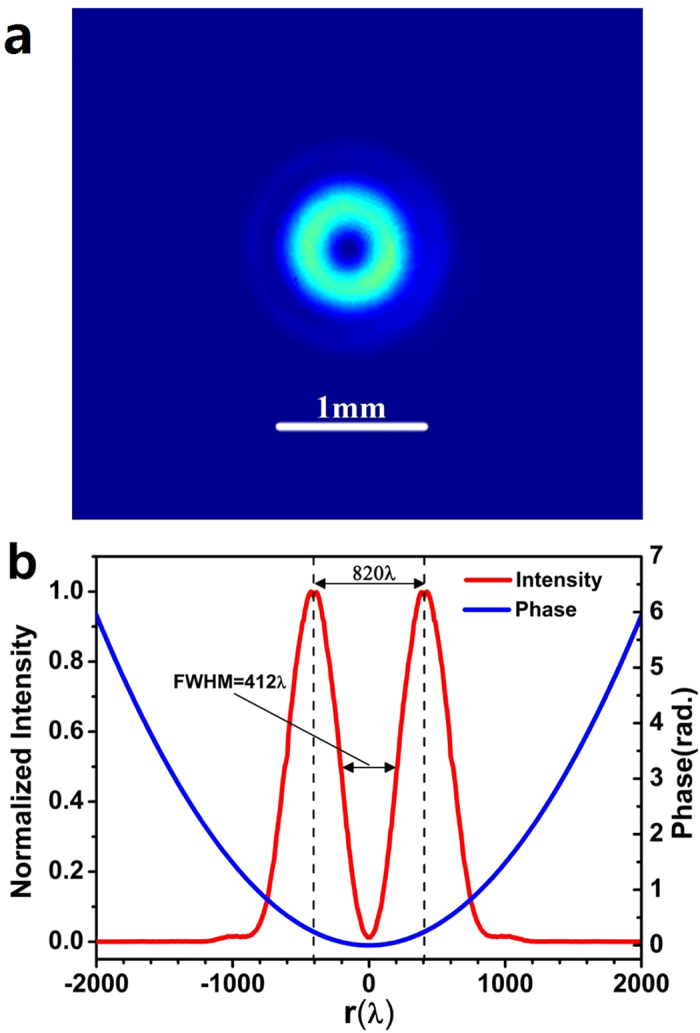
(**a**) The color map of the azimuthally polarized light intensity distribution on the lens incident surface; (**b**) the corresponding optical intensity and phase distribution in the radial direction.

**Figure 2:**
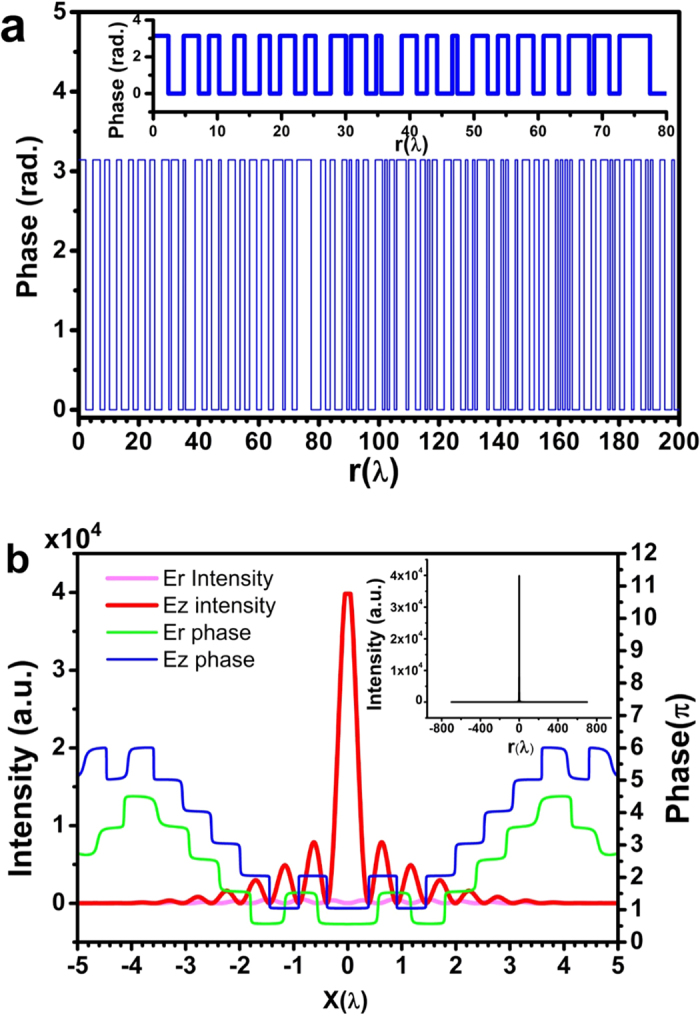
(**a**) The optimized phase distribution of the lens; (**b**) the intensity (red) and phase (blue) distribution of the longitudinal electrical component, and the intensity (pink) and phase (green) distribution of the radial electrical component on the focal plane. The inset plots the total optical intensity on the focal plane at z = 200λ.

To design the binary phase lens, the incident radially polarized light was first produced by illuminating an s-wave plate (Workshop of Photonics, Lithuania) with a normal incident linearly polarized Gaussian beam at a wavelength of 632.8 nm from a He-Ne laser. The resulting doughnut-shaped intensity profile was taken with a CCD camera, as shown in Fig. 1(a), which illustrates a Laguerre–Gaussian hollow ring structure with w_0_ = 331 μm and z = 276 mm. The corresponding phase distribution was deduced using equation 1, by fitting the amplitudes and phases of electrical fields on two measured planes perpendicular to the optical axis. Figure 2(b) gives the corresponding optical intensity and phase distribution along the radial direction, which shows a peak-peak diameter of 820λ and a hollow ring full width at half maximum (FWHM) of 412λ.

According to the obtained electrical field distribution, a binary phase lens consisting of concentric rings was optimized for a normally incident radially polarized wave at wavelength λ = 632.8 nm, by utilizing the genetic algorithm^33^ and the vectorial angular spectrum formulas given in equations (2). The radius of the lens is *R* = 500λ, its focal length is *f* = 200λ, and its NA is sin[arctan(*f*/*R*)] = 0.93. The lens diffraction limit (0.5λ/NA) and super-oscillation criterion (0.38λ/NA)^34^ are 0.54λ and 0.41λ respectively. The width of the ring is an integer multiple of *T* = 500 nm. The focal spot was designed with FWHM of 240 nm (0.38λ), which is smaller than the diffraction limit of 0.54λ and the super-oscillation criterion of 0.41λ. Figure 2(a) gives the optimized phase spatial distribution on the planar lens, and its inset shows a zoom-in plot of the phase distribution. The details of the phase distribution can be found in the supplement. The theoretically optimized electrical field distribution on the focal plane is depicted for E_r_ and E_z_, respectively, with respect to the radial coordinate, in Fig. 2(b). As expected, the longitudinal component E_z_ makes the major contribution to the formation of the focal spot, and its peak intensity is about 74 times greater than the maximum intensity of the radial component E_r_. Sharp phase inversion was observed at those points where the intensity of each electrical component was zero, which is the unique property of a super-oscillatory electrical field. The sidelobe intensity on the focal plane was found to be less than 20% of the central lobe peak intensity, resulting in a very clear field of view in the area of concern of [−700λ, +700λ], as depicted in the inset of Fig. 2(b).

### Numerical simulation of the longitudinally polarized spot generation

**Figure 3:**
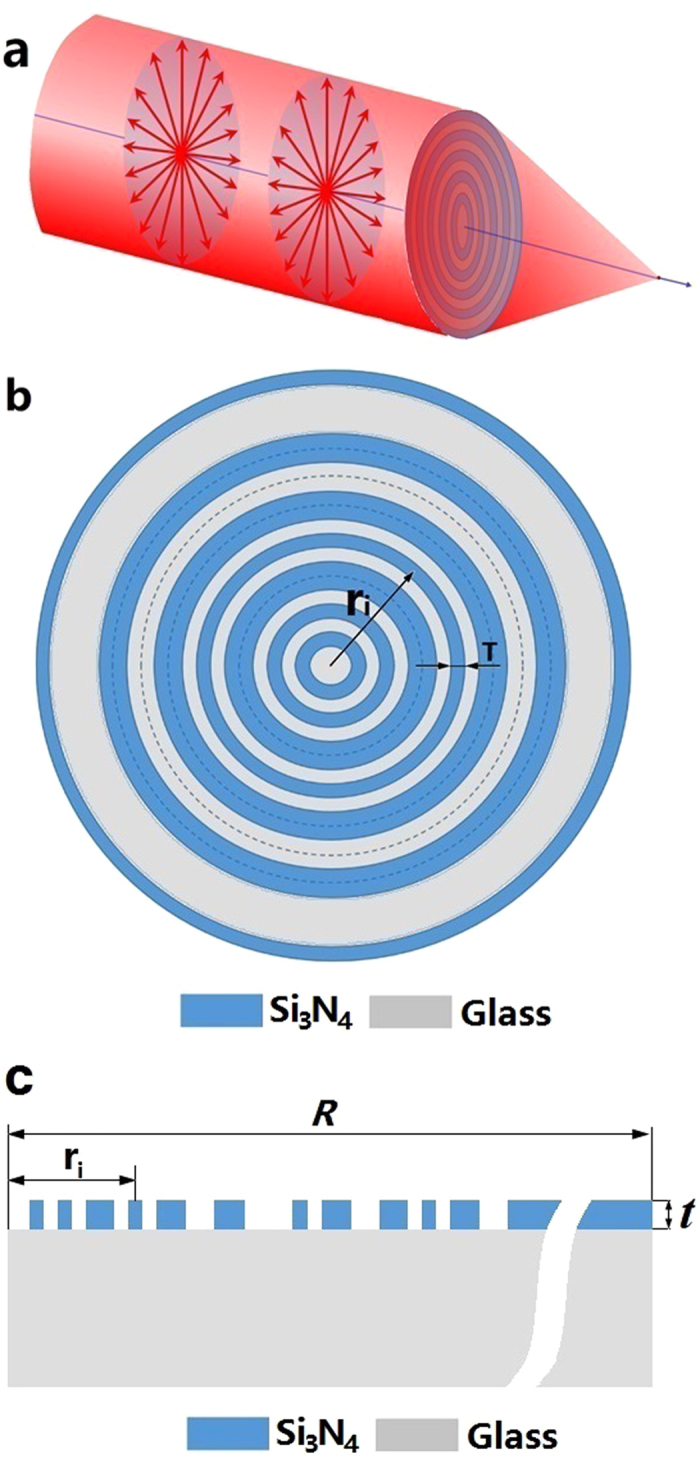
(**a**) Generation of a sub-diffraction longitudinal spot by focusing the radially polarized wave with a planar binary phase lens; (**b**) the micro lens structure; (**c**) the micro lens physical model for COMSOL numerical simulation.

To realize the binary phase lens, concentric dielectric ring layers were employed to achieve the desired phase distribution. Figure 3(a) shows the working principle of the lens. As shown in Fig. 3(b), the binary lens consists of a series of concentric Si_3_N_4_ rings grown on the top of a glass substrate. The thickness and the minimum width of the rings are *t* and *T*, respectively. The binary phase of 0 and π was achieved by controlling the thickness of the Si_3_N_4_ layer *t*, which is determined by *t* = *φλ*/2π(*n*_Si3N4_−1), where *φ* corresponds to the desired phase value of 0 or π, and *n*_Si3N4_ is the refractive index of Si_3_N_4_.

**Figure 4:**
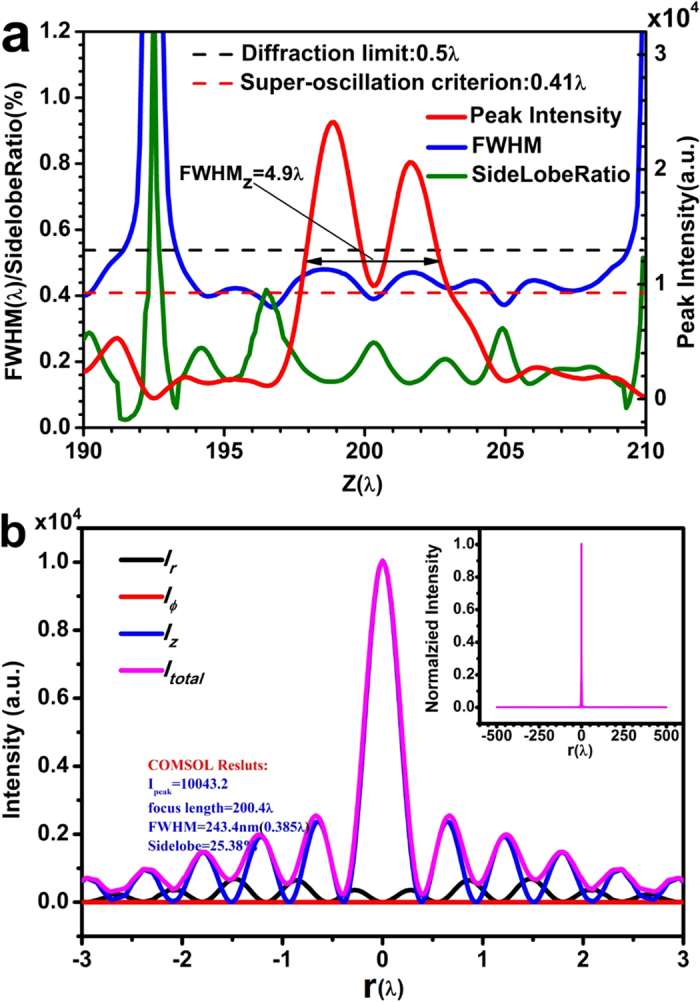
COMSOL numerical simulation results. (**a**) the spot FWHM (blue), sidelobe ratio (green), and intensity (red) distribution on the optical axis near the focal point, where the black dashed line and red dashed line indicate the diffraction limit and super-oscillatory criterion, respectively; (**b**) the total optical intensity (pink), and the intensities of radial (black), azimuthal (red) and longitudinal (blue) components on the focal plane at z = 200.4λ.

Numerical simulation was conducted for a dielectric ring binary phase lens based on the phase distribution described in Fig. 2(a), using COMSOL Multiphysics software. Utilizing the circular symmetry of the radially polarized incident wave and the lens, the 3-dimentional model can be simplified into a 2-dimentional model. As shown in Fig. 3(c), a 2-dimentional model was constructed in the positive half XZ plane (X > 0), which consists of ring structures obtained during lens design (as presented in the supplementary materials). In the physical model, the refractive index of Si_3_N_4_ is set at 1.91, and therefore the thicknesses corresponding to phase 0 and π are *t *= 0 and 348 nm, respectively. In the simulation, scattering boundary condition and perfectly matched layer was used to avoid unphysical reflection by the boundary. Figure 4(a) plots the peak intensity (red), spot transverse FWHM (blue), and sidelobe ratio (green) (the ratio of the maximum sidelobe intensity to the central lobe intensity) along the optical axis near the designed focal point at z = 200λ. As illustrated by the red curve, there is a double-hump-shaped spot with a longitudinal FWHM of FWHM_z_ = 5λ in the propagation direction. The two humps are located at z = 198.9λ and 201.6λ on the optical axis. The intensity at the valley is only 41% of the spot peak intensity. It is interesting to find that this valley point is located at z = 200.3λ and has the minimum transverse FWHM of 0.39λ, which are close to the designed focal length of 200λ and FWHM of 0.38, respectively. In the small region of [200λ, 200.6λ] around the focal point z = 200.3λ, the transverse FWHM is below the super-oscillatory criterion. According to the blue curve, in the area of [197.8λ, 202.8λ] in the propagation direction, as indicated by the arrows, the transverse FWHM of the spot is less than the diffraction limit, resulting in a sub-diffraction longitudinally polarized optical needle with a length of 5λ. It was found that, as shown in the green curve, the transverse sidelobe ratio of the whole focal spot in the area of [197.8λ, 202.8λ] is less than 25.8%. The total optical intensity and optical intensity of each electrical component at z = 200.3λ are plotted in Fig. 4(b). As expected, the azimuth polarization (red) is zero, and the radial polarization has intensity 64 times smaller than that of the longitudinal polarization, similar to above design. The inset of Fig. 3(b) depicts the normalized intensity distribution on the focal plane at z = 200.3λ, which shows a clear field of view within the radius of 500λ, agreeing with the theoretical design.

### Binary phase lens fabrication

**Figure 5:**
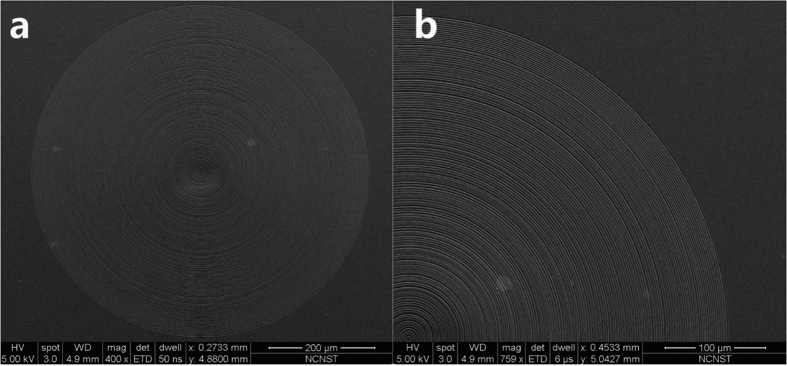
SEM images of the microlens based on binary phase mask.

Following the above design, a binary phase lens was fabricated on a 500-μm-thick sapphire substrate using electron-beam lithography and dry etching. The Si_3_N_4_ layer was first deposited on the substrate by plasma-enhanced chemical vapor deposition, and the Si_3_N_4_ layer was measured by an ellipsometry, which gave a refractive index of 1.91. The dielectric layer thickness was about 348 nm, corresponding to the relative phase change of π. Figure 5 shows the SEM images of the lens.

### Experimental methods

To experimentally generate the sub-diffraction longitudinally polarized spot, a radially polarized light was first produced with a linearly polarized Gaussian beam at a wavelength of 632.8 nm by an s-wave plate (Workshop of Photonics, Lithuania). The beam profile is presented in Fig. 1. The power of the generated radially polarized light is about 1.4 mW. In the experiment, the radially polarized wave normally illuminated on the lens from the substrate side. The diffracted optical intensity distribution behind the lens was obtained with a tapered optical fiber probe (CFN-100 from Nanonics Imaging, Ltd., Israel) mounted on a 3-D piezo nanopositioner (P-561.3CD from Physik Instrumente GmbH & Co., Germany). The probe tip diameter was about 100 nm. The spatial resolution and scanning range of the nanopositioner were about 10 nm and 100 μm, respectively, for each of the x-, y-, and z-axes. The collected photons were counted with a single photon detector (SPCM50A/M from Thorlabs, Inc., USA).

## Results and Discussion

**Figure 6:**
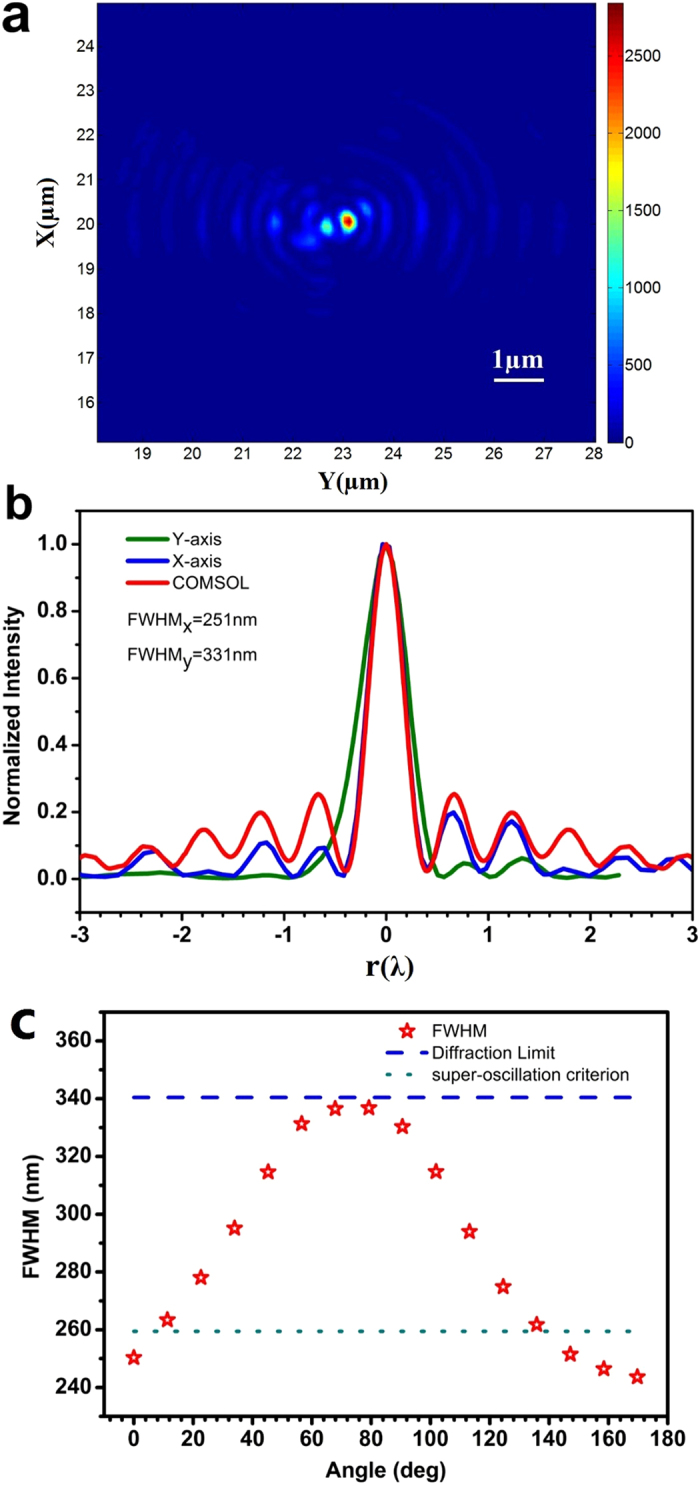
(**a**) Color map of the focal plane intensity distribution; (**b**) intensity distribution along the x-axis (green) and y-axis (blue), where the numerical simulation result of the optical intensity (red) is also depicted for comparison; (**c**) the focal spot FWHM in different directions, where the y-axis value is the angle between the direction and the positive y-axis, and the dashed and dotted lines denote the diffraction limit (341 nm) and super-oscillation criterion (259 nm) of the lens, respectively.

Figure 6(a) illustrates the in-plane optical intensity distribution at z = 201.4λ; except for a single weak spot near the central focal point, no large sidelobe was found in the 10 μm × 10 μm scanned square area. In Fig. 6(b), the beam transverse profiles are plotted against the x-axis (blue) and y-axis (green), respectively, and the numerical simulation result (red) is also depicted for comparison. It was found that the FWHM is 331 nm and 251 nm in the x-axis and y-axis, respectively. The FWHM in the y-axis is smaller than the super-oscillatory criterion of 259 nm, and is close to the designed value of 240 nm, while the FWHM in the x-axis is larger than the super-oscillatory criterion, but still smaller than the diffraction limit of 341 nm. For a better evaluation of the spot size, we measured the FWHM across the spot center in different directions, as plotted in Fig. 6(c). All the FWHM values are smaller than the diffraction limit, and four of them are below the super-oscillatory criterion. The average FWHM is 289 nm. This asymmetry is believed to be caused by errors in the alignment of the three optical axes of the laser beam, s-wave plate and testing sample in the experiment.

**Figure 7:**
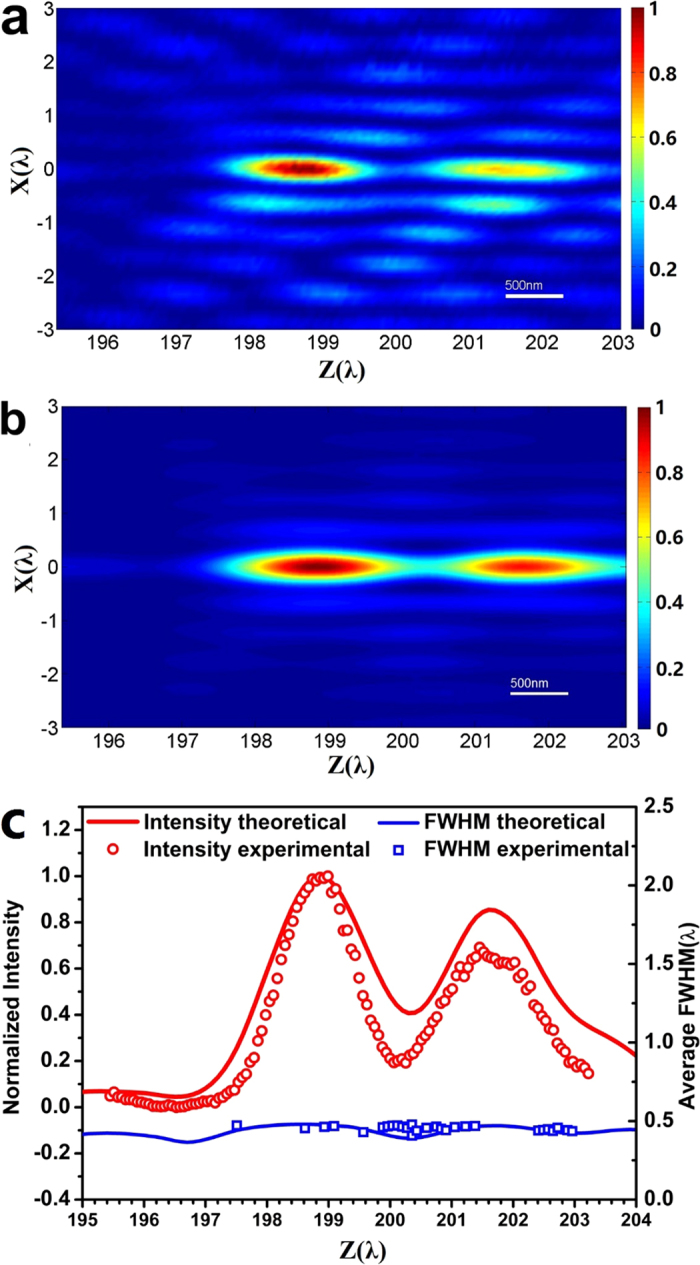
Color maps of the optical intensity on the y-z plane near the focal point. (**a**) the experimental result; (**b**) the numerical simulation; (**c**) optical intensities on the optical axis (z-axis) near the focal point: numerical simulation (red curve) and experimental result (red circles); and the transverse FWHM on the optical axis near the focal point: numerical simulation (blue curve) and experimental result (blue squares).

Figure 7(a) illustrates the experimentally obtained optical intensity distribution in the y-z plane near the designed focal spot position. Along the optical axis, two connected focal spots were found, corresponding to the double humps located at z = 198.9λ and 201.6λ, as shown in Fig. 4(a). For comparison, the optical intensity in the same area obtained by COMSOL numerical simulation is plotted in Fig. 7(b), which shows good similarity to the experimental result. The optical intensity along the optical axis is plotted in Fig. 7(c); the COMSOL numerical simulation of the y-z plane optical intensity (red curve) is also plotted, and is similar to the experimental data (red circles). The average transverse FWHM is also plotted for experimental (blue squares) and numerical simulation (blue curve) respectively. In range from z = 197.4λ to 203.2λ, the average FWHM is less than the diffraction limit 0.54λ, resulting in a 5λ-long sub-diffraction optical needle with longitudinal polarization. Although sub-diffraction optical needles with linear polarization^35^ and circular polarizaiton^20^,^24^ have been experimentally demonstrated previously, the optical needle reported here has a unique longitudinally polarization along the optical axis.

## Conclusions

In conclusion, we have proposed and experimentally demonstrated the generation of a sub-diffraction longitudinally polarized spot by focusing a radially polarized light using a binary phase lens. This lens has a long focal length of about 200λ and NA of 0.93. The designed focal spot has an FWHM of 240 nm, which is below the super-oscillatory criterion of 0.41λ. The experimental results show a focal spot with average FWHM of about 289 nm, smaller than the diffraction limit of 342 nm. The spot longitudinal size is about 5λ in the propagation direction, leading to a sub-diffraction optical needle with longitudinal polarization. Although high-NA conventional lenses have been suggested to generate longitudinally polarized light, here we see that they can also be created theoretically and experimentally by using a pure binary phase lens alone. Not only is the sub-diffraction longitudinally polarized needle important for super-resolution microscopy, but it might also find its applications in optical tweezers, Raman spectroscopy, and optical data storage. This approach is also applicable to other optical spectrum ranges. A combination of focusing and polarization conversion in a single phase lens is expected to further reduce the size of a longitudinally polarized spot in the experiment, for less difficulty in optical alignment.

## Additional Information

**How to cite this article**: Yu, A.- *et al*. Creation of Sub-diffraction Longitudinally Polarized Spot by Focusing Radially Polarized Light with Binary Phase Lens. *Sci. Rep.* **6**, 38859; doi: 10.1038/srep38859 (2016).

**Publisher's note:** Springer Nature remains neutral with regard to jurisdictional claims in published maps and institutional affiliations.

## Supplementary Material

Supplementary Information
