# Supplementary material for: Creation of Sub-diffraction Longitudinally Polarized Spot by Focusing Radially Polarized Light with Binary Phase Lens
Source: Sci Rep. 2016 Dec 12;6:38859. doi: 10.1038/srep38859 (PMC5150649; doi:10.1038/srep38859)
Supplement: Supplementary Information [file srep38859-s1.doc]

Creation of Sub-diffraction Longitudinally Polarized Spot by Focusing Radially Polarized Light with Binary Phase Lens

An-ping Yu^1,*^, Gang Chen^1, †,*^, Zhi-hai Zhang^1,*^, Zhong-quan Wen^1,††,*^, Lu-ru Dai ^2,†††, *^, Kun Zhang^1,2,*^ , Sen-ling Jiang^1^, Zhi-xiang Wu^1^, Yu-yan Li^1^, Chang-tao Wang^3^, Xian-gang Luo^3^

^1^Key Laboratory of Optoelectronic Technology and Systems (Chongqing University), Ministry of Education, and Key Disciplines Lab of Novel Micro-nano Devices and System Technology, Chongqing University,173 Shazheng Street, Shapingba, Chongqing 400044, China. ^2^National Center for Nanoscience and Technology, No.11 Zhong Guan CunBei Yi Tiao, Beijing 100190, China. ^3^State Key Laboratory of Optical Technologies on Nano-Fabrication and Micro-Engineering, Institute of Optics and Electronics, Chinese Academy of Science, P. R. Box 350, Chengdu 610209, China

**Supplementary**

**Table 1. the geometry of the micro-lens (T=500 nm)**

| i | Ri  (nm) | t_Si3N4_  (nm) | i | Ri  (nm) | t_Si3N4_  (nm) | i | Ri  (nm) | t_Si3N4_  (nm) |
| --- | --- | --- | --- | --- | --- | --- | --- | --- |
| 0 | 0 | 348 | 48 | 24000 | 0 | 96 | 48000 | 348 |
| 1 | 500 | 348 | 49 | 24500 | 0 | 97 | 48500 | 348 |
| 2 | 1000 | 348 | 50 | 25000 | 348 | 98 | 49000 | 348 |
| 3 | 1500 | 348 | 51 | 25500 | 348 | 99 | 49500 | 0 |
| 4 | 2000 | 0 | 52 | 26000 | 348 | 100 | 50000 | 0 |
| 5 | 2500 | 0 | 53 | 26500 | 0 | 101 | 50500 | 0 |
| 6 | 3000 | 0 | 54 | 27000 | 0 | 102 | 51000 | 0 |
| 7 | 3500 | 348 | 55 | 27500 | 348 | 103 | 51500 | 348 |
| 8 | 4000 | 348 | 56 | 28000 | 348 | 104 | 52000 | 348 |
| 9 | 4500 | 348 | 57 | 28500 | 0 | 105 | 52500 | 0 |
| 10 | 5000 | 0 | 58 | 29000 | 0 | 106 | 53000 | 0 |
| 11 | 5500 | 0 | 59 | 29500 | 0 | 107 | 53500 | 348 |
| 12 | 6000 | 348 | 60 | 30000 | 348 | 108 | 54000 | 348 |
| 13 | 6500 | 348 | 61 | 30500 | 0 | 109 | 54500 | 0 |
| 14 | 7000 | 0 | 62 | 31000 | 0 | 110 | 55000 | 0 |
| 15 | 7500 | 0 | 63 | 31500 | 0 | 111 | 55500 | 0 |
| 16 | 8000 | 0 | 64 | 32000 | 348 | 112 | 56000 | 348 |
| 17 | 8500 | 348 | 65 | 32500 | 348 | 113 | 56500 | 348 |
| 18 | 9000 | 348 | 66 | 33000 | 348 | 114 | 57000 | 0 |
| 19 | 9500 | 0 | 67 | 33500 | 0 | 115 | 57500 | 348 |
| 20 | 10000 | 0 | 68 | 34000 | 0 | 116 | 58000 | 0 |
| 21 | 10500 | 0 | 69 | 34500 | 348 | 117 | 58500 | 0 |
| 22 | 11000 | 348 | 70 | 35000 | 348 | 118 | 59000 | 348 |
| 23 | 11500 | 348 | 71 | 35500 | 0 | 119 | 59500 | 0 |
| 24 | 12000 | 0 | 72 | 36000 | 0 | 120 | 60000 | 0 |
| 25 | 12500 | 0 | 73 | 36500 | 348 | 121 | 60500 | 348 |
| 26 | 13000 | 348 | 74 | 37000 | 348 | 122 | 61000 | 348 |
| 27 | 13500 | 348 | 75 | 37500 | 348 | 123 | 61500 | 0 |
| 28 | 14000 | 348 | 76 | 38000 | 0 | 124 | 62000 | 0 |
| 29 | 14500 | 0 | 77 | 38500 | 0 | 125 | 62500 | 0 |
| 30 | 15000 | 0 | 78 | 39000 | 348 | 126 | 63000 | 348 |
| 31 | 15500 | 348 | 79 | 39500 | 348 | 127 | 63500 | 348 |
| 32 | 16000 | 348 | 80 | 40000 | 348 | 128 | 64000 | 348 |
| 33 | 16500 | 0 | 81 | 40500 | 0 | 129 | 64500 | 0 |
| 34 | 17000 | 0 | 82 | 41000 | 0 | 130 | 65000 | 348 |
| 35 | 17500 | 0 | 83 | 41500 | 348 | 131 | 65500 | 0 |
| 36 | 18000 | 348 | 84 | 42000 | 348 | 132 | 66000 | 348 |
| 37 | 18500 | 348 | 85 | 42500 | 348 | 133 | 66500 | 348 |
| 38 | 19000 | 348 | 86 | 43000 | 348 | 134 | 67000 | 0 |
| 39 | 19500 | 0 | 87 | 43500 | 0 | 135 | 67500 | 348 |
| 40 | 20000 | 348 | 88 | 44000 | 348 | 136 | 68000 | 348 |
| 41 | 20500 | 348 | 89 | 44500 | 348 | 137 | 68500 | 348 |
| 42 | 21000 | 348 | 90 | 45000 | 348 | 138 | 69000 | 348 |
| 43 | 21500 | 0 | 91 | 45500 | 0 | 139 | 69500 | 0 |
| 44 | 22000 | 0 | 92 | 46000 | 0 | 140 | 70000 | 348 |
| 45 | 22500 | 348 | 93 | 46500 | 348 | 141 | 70500 | 348 |
| 46 | 23000 | 0 | 94 | 47000 | 348 | 142 | 71000 | 348 |
| 47 | 23500 | 0 | 95 | 47500 | 348 | 143 | 71500 | 0 |

| i | Ri  (nm) | tSi3N4  (nm) | i | Ri  (nm) | tSi3N4  (nm) | i | Ri  (nm) | tSi3N4  (nm) |
| --- | --- | --- | --- | --- | --- | --- | --- | --- |
| 144 | 72000 | 0 | 192 | 96000 | 0 | 240 | 120000 | 348 |
| 145 | 72500 | 348 | 193 | 96500 | 348 | 241 | 120500 | 0 |
| 146 | 73000 | 348 | 194 | 97000 | 348 | 242 | 121000 | 348 |
| 147 | 73500 | 0 | 195 | 97500 | 0 | 243 | 121500 | 0 |
| 148 | 74000 | 348 | 196 | 98000 | 0 | 244 | 122000 | 0 |
| 149 | 74500 | 0 | 197 | 98500 | 348 | 245 | 122500 | 0 |
| 150 | 75000 | 348 | 198 | 99000 | 348 | 246 | 123000 | 348 |
| 151 | 75500 | 348 | 199 | 99500 | 0 | 247 | 123500 | 348 |
| 152 | 76000 | 0 | 200 | 100000 | 0 | 248 | 124000 | 0 |
| 153 | 76500 | 0 | 201 | 100500 | 0 | 249 | 124500 | 0 |
| 154 | 77000 | 0 | 202 | 101000 | 348 | 250 | 125000 | 0 |
| 155 | 77500 | 348 | 203 | 101500 | 0 | 251 | 125500 | 348 |
| 156 | 78000 | 348 | 204 | 102000 | 348 | 252 | 126000 | 0 |
| 157 | 78500 | 348 | 205 | 102500 | 0 | 253 | 126500 | 0 |
| 158 | 79000 | 0 | 206 | 103000 | 348 | 254 | 127000 | 0 |
| 159 | 79500 | 348 | 207 | 103500 | 0 | 255 | 127500 | 348 |
| 160 | 80000 | 348 | 208 | 104000 | 348 | 256 | 128000 | 348 |
| 161 | 80500 | 348 | 209 | 104500 | 0 | 257 | 128500 | 0 |
| 162 | 81000 | 0 | 210 | 105000 | 0 | 258 | 129000 | 0 |
| 163 | 81500 | 0 | 211 | 105500 | 0 | 259 | 129500 | 0 |
| 164 | 82000 | 348 | 212 | 106000 | 348 | 260 | 130000 | 348 |
| 165 | 82500 | 0 | 213 | 106500 | 348 | 261 | 130500 | 348 |
| 166 | 83000 | 0 | 214 | 107000 | 0 | 262 | 131000 | 348 |
| 167 | 83500 | 348 | 215 | 107500 | 0 | 263 | 131500 | 0 |
| 168 | 84000 | 0 | 216 | 108000 | 0 | 264 | 132000 | 348 |
| 169 | 84500 | 348 | 217 | 108500 | 348 | 265 | 132500 | 348 |
| 170 | 85000 | 348 | 218 | 109000 | 348 | 266 | 133000 | 348 |
| 171 | 85500 | 348 | 219 | 109500 | 0 | 267 | 133500 | 0 |
| 172 | 86000 | 348 | 220 | 110000 | 0 | 268 | 134000 | 0 |
| 173 | 86500 | 0 | 221 | 110500 | 348 | 269 | 134500 | 348 |
| 174 | 87000 | 348 | 222 | 111000 | 348 | 270 | 135000 | 348 |
| 175 | 87500 | 348 | 223 | 111500 | 348 | 271 | 135500 | 348 |
| 176 | 88000 | 0 | 224 | 112000 | 0 | 272 | 136000 | 0 |
| 177 | 88500 | 0 | 225 | 112500 | 348 | 273 | 136500 | 0 |
| 178 | 89000 | 0 | 226 | 113000 | 0 | 274 | 137000 | 348 |
| 179 | 89500 | 348 | 227 | 113500 | 348 | 275 | 137500 | 348 |
| 180 | 90000 | 0 | 228 | 114000 | 348 | 276 | 138000 | 348 |
| 181 | 90500 | 348 | 229 | 114500 | 0 | 277 | 138500 | 0 |
| 182 | 91000 | 0 | 230 | 115000 | 0 | 278 | 139000 | 348 |
| 183 | 91500 | 0 | 231 | 115500 | 348 | 279 | 139500 | 348 |
| 184 | 92000 | 0 | 232 | 116000 | 348 | 280 | 140000 | 0 |
| 185 | 92500 | 348 | 233 | 116500 | 348 | 281 | 140500 | 348 |
| 186 | 93000 | 0 | 234 | 117000 | 0 | 282 | 141000 | 0 |
| 187 | 93500 | 0 | 235 | 117500 | 348 | 283 | 141500 | 348 |
| 188 | 94000 | 348 | 236 | 118000 | 348 | 284 | 142000 | 348 |
| 189 | 94500 | 348 | 237 | 118500 | 348 | 285 | 142500 | 348 |
| 190 | 95000 | 348 | 238 | 119000 | 0 | 286 | 143000 | 0 |
| 191 | 95500 | 0 | 239 | 119500 | 0 | 287 | 143500 | 0 |

| i | Ri  (nm) | t_Si3N4_  (nm) | i | Ri  (nm) | t_Si3N4_  (nm) | i | Ri  (nm) | t_Si3N4_  (nm) |
| --- | --- | --- | --- | --- | --- | --- | --- | --- |
| 288 | 144000 | 348 | 337 | 168500 | 348 | 386 | 193000 | 0 |
| 289 | 144500 | 348 | 338 | 169000 | 0 | 387 | 193500 | 0 |
| 290 | 145000 | 0 | 339 | 169500 | 0 | 388 | 194000 | 348 |
| 291 | 145500 | 0 | 340 | 170000 | 0 | 389 | 194500 | 348 |
| 292 | 146000 | 348 | 341 | 170500 | 348 | 390 | 195000 | 348 |
| 293 | 146500 | 348 | 342 | 171000 | 348 | 391 | 195500 | 0 |
| 294 | 147000 | 348 | 343 | 171500 | 0 | 392 | 196000 | 0 |
| 295 | 147500 | 0 | 344 | 172000 | 0 | 393 | 196500 | 348 |
| 296 | 148000 | 0 | 345 | 172500 | 348 | 394 | 197000 | 348 |
| 297 | 148500 | 0 | 346 | 173000 | 348 | 395 | 197500 | 0 |
| 298 | 149000 | 348 | 347 | 173500 | 348 | 396 | 198000 | 0 |
| 299 | 149500 | 348 | 348 | 174000 | 0 | 397 | 198500 | 0 |
| 300 | 150000 | 0 | 349 | 174500 | 0 | 398 | 199000 | 348 |
| 301 | 150500 | 0 | 350 | 175000 | 348 | 399 | 199500 | 348 |
| 302 | 151000 | 0 | 351 | 175500 | 348 | 400 | 200000 | 0 |
| 303 | 151500 | 348 | 352 | 176000 | 0 | 401 | 200500 | 0 |
| 304 | 152000 | 348 | 353 | 176500 | 0 | 402 | 201000 | 348 |
| 305 | 152500 | 0 | 354 | 177000 | 0 | 403 | 201500 | 348 |
| 306 | 153000 | 0 | 355 | 177500 | 348 | 404 | 202000 | 348 |
| 307 | 153500 | 0 | 356 | 178000 | 348 | 405 | 202500 | 348 |
| 308 | 154000 | 348 | 357 | 178500 | 0 | 406 | 203000 | 0 |
| 309 | 154500 | 348 | 358 | 179000 | 0 | 407 | 203500 | 0 |
| 310 | 155000 | 0 | 359 | 179500 | 0 | 408 | 204000 | 348 |
| 311 | 155500 | 348 | 360 | 180000 | 348 | 409 | 204500 | 348 |
| 312 | 156000 | 348 | 361 | 180500 | 348 | 410 | 205000 | 0 |
| 313 | 156500 | 348 | 362 | 181000 | 0 | 411 | 205500 | 0 |
| 314 | 157000 | 348 | 363 | 181500 | 0 | 412 | 206000 | 348 |
| 315 | 157500 | 0 | 364 | 182000 | 348 | 413 | 206500 | 348 |
| 316 | 158000 | 348 | 365 | 182500 | 348 | 414 | 207000 | 348 |
| 317 | 158500 | 348 | 366 | 183000 | 348 | 415 | 207500 | 0 |
| 318 | 159000 | 348 | 367 | 183500 | 0 | 416 | 208000 | 0 |
| 319 | 159500 | 348 | 368 | 184000 | 0 | 417 | 208500 | 348 |
| 320 | 160000 | 0 | 369 | 184500 | 348 | 418 | 209000 | 348 |
| 321 | 160500 | 0 | 370 | 185000 | 348 | 419 | 209500 | 0 |
| 322 | 161000 | 348 | 371 | 185500 | 348 | 420 | 210000 | 0 |
| 323 | 161500 | 0 | 372 | 186000 | 0 | 421 | 210500 | 348 |
| 324 | 162000 | 0 | 373 | 186500 | 0 | 422 | 211000 | 348 |
| 325 | 162500 | 0 | 374 | 187000 | 0 | 423 | 211500 | 348 |
| 326 | 163000 | 348 | 375 | 187500 | 348 | 424 | 212000 | 0 |
| 327 | 163500 | 348 | 376 | 188000 | 348 | 425 | 212500 | 0 |
| 328 | 164000 | 348 | 377 | 188500 | 0 | 426 | 213000 | 348 |
| 329 | 164500 | 0 | 378 | 189000 | 348 | 427 | 213500 | 348 |
| 330 | 165000 | 0 | 379 | 189500 | 348 | 428 | 214000 | 0 |
| 331 | 165500 | 348 | 380 | 190000 | 348 | 429 | 214500 | 0 |
| 332 | 166000 | 348 | 381 | 190500 | 0 | 430 | 215000 | 0 |
| 333 | 166500 | 348 | 382 | 191000 | 0 | 431 | 215500 | 348 |
| 334 | 167000 | 0 | 383 | 191500 | 0 | 432 | 216000 | 348 |
| 335 | 167500 | 0 | 384 | 192000 | 348 | 433 | 216500 | 348 |
| 336 | 168000 | 348 | 385 | 192500 | 348 | 434 | 217000 | 0 |

| i | Ri  (nm) | t_Si3N4_  (nm) | i | Ri  (nm) | t_Si3N4_  (nm) | i | Ri  (nm) | t_Si3N4_  (nm) |
| --- | --- | --- | --- | --- | --- | --- | --- | --- |
| 435 | 217500 | 0 | 484 | 242000 | 348 | 533 | 266500 | 348 |
| 436 | 218000 | 348 | 485 | 242500 | 348 | 534 | 267000 | 0 |
| 437 | 218500 | 348 | 486 | 243000 | 0 | 535 | 267500 | 0 |
| 438 | 219000 | 0 | 487 | 243500 | 0 | 536 | 268000 | 348 |
| 439 | 219500 | 0 | 488 | 244000 | 348 | 537 | 268500 | 348 |
| 440 | 220000 | 348 | 489 | 244500 | 348 | 538 | 269000 | 0 |
| 441 | 220500 | 348 | 490 | 245000 | 0 | 539 | 269500 | 0 |
| 442 | 221000 | 348 | 491 | 245500 | 0 | 540 | 270000 | 348 |
| 443 | 221500 | 0 | 492 | 246000 | 348 | 541 | 270500 | 348 |
| 444 | 222000 | 0 | 493 | 246500 | 348 | 542 | 271000 | 348 |
| 445 | 222500 | 348 | 494 | 247000 | 348 | 543 | 271500 | 348 |
| 446 | 223000 | 348 | 495 | 247500 | 348 | 544 | 272000 | 0 |
| 447 | 223500 | 348 | 496 | 248000 | 0 | 545 | 272500 | 0 |
| 448 | 224000 | 0 | 497 | 248500 | 0 | 546 | 273000 | 348 |
| 449 | 224500 | 0 | 498 | 249000 | 348 | 547 | 273500 | 348 |
| 450 | 225000 | 0 | 499 | 249500 | 348 | 548 | 274000 | 0 |
| 451 | 225500 | 348 | 500 | 250000 | 0 | 549 | 274500 | 0 |
| 452 | 226000 | 348 | 501 | 250500 | 0 | 550 | 275000 | 348 |
| 453 | 226500 | 0 | 502 | 251000 | 348 | 551 | 275500 | 348 |
| 454 | 227000 | 0 | 503 | 251500 | 348 | 552 | 276000 | 0 |
| 455 | 227500 | 348 | 504 | 252000 | 0 | 553 | 276500 | 0 |
| 456 | 228000 | 348 | 505 | 252500 | 0 | 554 | 277000 | 348 |
| 457 | 228500 | 0 | 506 | 253000 | 0 | 555 | 277500 | 348 |
| 458 | 229000 | 0 | 507 | 253500 | 0 | 556 | 278000 | 0 |
| 459 | 229500 | 348 | 508 | 254000 | 348 | 557 | 278500 | 348 |
| 460 | 230000 | 0 | 509 | 254500 | 348 | 558 | 279000 | 0 |
| 461 | 230500 | 348 | 510 | 255000 | 0 | 559 | 279500 | 0 |
| 462 | 231000 | 348 | 511 | 255500 | 0 | 560 | 280000 | 348 |
| 463 | 231500 | 0 | 512 | 256000 | 348 | 561 | 280500 | 348 |
| 464 | 232000 | 0 | 513 | 256500 | 348 | 562 | 281000 | 0 |
| 465 | 232500 | 348 | 514 | 257000 | 0 | 563 | 281500 | 0 |
| 466 | 233000 | 348 | 515 | 257500 | 0 | 564 | 282000 | 348 |
| 467 | 233500 | 0 | 516 | 258000 | 348 | 565 | 282500 | 348 |
| 468 | 234000 | 0 | 517 | 258500 | 348 | 566 | 283000 | 0 |
| 469 | 234500 | 348 | 518 | 259000 | 348 | 567 | 283500 | 0 |
| 470 | 235000 | 348 | 519 | 259500 | 348 | 568 | 284000 | 348 |
| 471 | 235500 | 348 | 520 | 260000 | 0 | 569 | 284500 | 348 |
| 472 | 236000 | 0 | 521 | 260500 | 0 | 570 | 285000 | 0 |
| 473 | 236500 | 0 | 522 | 261000 | 348 | 571 | 285500 | 348 |
| 474 | 237000 | 348 | 523 | 261500 | 348 | 572 | 286000 | 0 |
| 475 | 237500 | 348 | 524 | 262000 | 0 | 573 | 286500 | 0 |
| 476 | 238000 | 0 | 525 | 262500 | 0 | 574 | 287000 | 0 |
| 477 | 238500 | 0 | 526 | 263000 | 348 | 575 | 287500 | 348 |
| 478 | 239000 | 348 | 527 | 263500 | 348 | 576 | 288000 | 348 |
| 479 | 239500 | 348 | 528 | 264000 | 0 | 577 | 288500 | 0 |
| 480 | 240000 | 0 | 529 | 264500 | 348 | 578 | 289000 | 0 |
| 481 | 240500 | 0 | 530 | 265000 | 0 | 579 | 289500 | 348 |
| 482 | 241000 | 0 | 531 | 265500 | 348 | 580 | 290000 | 348 |
| 483 | 241500 | 0 | 532 | 266000 | 348 | 581 | 290500 | 0 |

| i | Ri  (nm) | t_Si3N4_  (nm) | i | Ri  (nm) | t_Si3N4_  (nm) | i | Ri  (nm) | t_Si3N4_  (nm) |
| --- | --- | --- | --- | --- | --- | --- | --- | --- |
| 582 | 291000 | 0 | 599 | 299500 | 0 | 616 | 308000 | 348 |
| 583 | 291500 | 348 | 600 | 300000 | 0 | 617 | 308500 | 348 |
| 584 | 292000 | 0 | 601 | 300500 | 348 | 618 | 309000 | 0 |
| 585 | 292500 | 0 | 602 | 301000 | 0 | 619 | 309500 | 0 |
| 586 | 293000 | 348 | 603 | 301500 | 0 | 620 | 310000 | 348 |
| 587 | 293500 | 0 | 604 | 302000 | 348 | 621 | 310500 | 0 |
| 588 | 294000 | 0 | 605 | 302500 | 348 | 622 | 311000 | 348 |
| 589 | 294500 | 348 | 606 | 303000 | 0 | 623 | 311500 | 348 |
| 590 | 295000 | 348 | 607 | 303500 | 0 | 624 | 312000 | 0 |
| 591 | 295500 | 0 | 608 | 304000 | 348 | 625 | 312500 | 0 |
| 592 | 296000 | 0 | 609 | 304500 | 348 | 626 | 313000 | 348 |
| 593 | 296500 | 348 | 610 | 305000 | 0 | 627 | 313500 | 348 |
| 594 | 297000 | 348 | 611 | 305500 | 0 | 628 | 314000 | 0 |
| 595 | 297500 | 0 | 612 | 306000 | 348 | 629 | 314500 | 0 |
| 596 | 298000 | 0 | 613 | 306500 | 348 | 630 | 315000 | 0 |
| 597 | 298500 | 348 | 614 | 307000 | 0 | 631 | 315500 | 348 |
| 598 | 299000 | 348 | 615 | 307500 | 0 | 632 | 316000 | 348 |
